# The New United States Pharmacopœia

**Published:** 1864-01

**Authors:** 


### SELECTED.

### THE NEW UNITED STATES PHARMACOPŒIA.

#### THE CHANGES THAT HAVE BEEN MADE, WITH THE REASONS FOR SO DOING.

Dr. E. R. Squibb, of Brooklyn, N. Y., a member of the Committee for Revision of the U. S. Pharmacopœia, reported at the Academy of Medicine that the work had been published, and made in substance the following statements in relation to the changes that had been made, together with the reasons for so doing.

*Weights and Measures.*—In the department of weights and measures the following changes have been made. The terms pound, drachm (for other than fluids), and scruple are disused, and all weights are expressed by the Troy ounce or grain. In writing for fluids the sign letter *f* should precede the ℥ or ʒ sign. The fluid measures are derived from the wine gallon. The term gallon is not used, its equivalent being expressed in pints. The use of the Arabic numerals is recommended as preferable to the Roman.

The Materia Medica list is divided into two sub-classes. I. Primary, and II. Secondary Lists. The first consists of those articles which are either of primary importance as remedies, or enter into the formulæ of the work, while the second class is formed of those which are of secondary importance and do not enter into the formulæ.

*Chromic acid* (acidum chromicum) is introduced as a valuable and self-limiting escharotic. Thus far it has been employed extensively in uterine diseases. It is applied through a speculum by a glass tube or a glass rod.

*Lactic acid* (acidum lacticum) is introduced for the purpose of making lactate of lime; and the *glacial phosphoric acid* is also placed in the primary list for the preparation of dilute phosphoric acid, a very valuable nervine tonic.

*Fusel oil* (Alcohol Amylicum) is introduced for making valerianic acid.

*Aloes* is divided into three varieties, I. Aloe Barbadensis, II. Aloe Capensis, and III. Aloe Socotrina, which is the best.

*Ammonia alum* (sulphate of aluminum and ammonia) is given for a choice as an ordinary astringent.

*Aqua Ammoniæ fortior.*—The term *aqua* is substituted for *liquor*, inasmuch as the former is intended to apply to a solution of volatile substances and the latter to fixed substances.

*Belladonna Radix* is added for the preparation of atropia.

*Bromine* is introduced to furnish the bromide of potassium, the reputed remedy for hospital gangrene.

*Chiretta* is added to the list of bitter tonics.

*Chloroformum Venale.*—Commercial chloroform is placed in this list to give an opportunity for prescribing the common article for external application, it being a great deal cheaper, and equally, for that purpose, as good as the pure.

*Cinchona Flava.*—The cinchonas are divided into C. flava and C. pallida, the flava (yellow) being the best.

*Fermentum* (yeast) is made officinal, that it may be written for in prescriptions; *gutta percha* is introduced for making the gutta percha collodion.

*The Butter of Cocoa* (Oleo theobromæ) is introduced as the best ingredient for suppositories.

*Opium.*—Opium must not be considered officinal unless it contain 7 per cent. of morphia.

*Potassæ Permanganas* is a most valuable disinfectant for carcinomatous growths, but hardly fit for anything else.

*Saccharum lactis* (sugar of milk) is introduced as a vehicle for alkaloids where it is necessary to divide very finely. It is very slowly soluble, and is by this means capable of disguising the taste of medicines.

*Scammony* must contain 75 per cent. of true resin to be officinal.

*Sinapis* (mustard) is divided into two varieties (white and black), both having different therapeutical properties.

*Whisky* (spiritus frumenti), and bay rum (spiritus myricæ) are officinal preparations. The former should contain 48 to 56 per cent. of alcohol, be free from odor, and be at least two years old.

*Port and Sherry Wines* are also officinal, the former is named Vinum Portense, and the latter Vinum Xericum.

*Sulphur.*—Sublimed sulphur is introduced to be used as an ingredient for ointments in cutaneous affections, inasmuch as it contains the sulphurous and sulphuric acid, which the washed rotund sulphur does not. This latter is used internally.

*Alcohol Fortius* (stronger alcohol) was introduced as a solvent for iodine and like substances.

*Alcohol Dilutum* is the stronger alcohol diluted one-half with water.

*Aurantii flores* is used for making the orange flower water, a useful substitute for the ordinary rose water.

*Cadmium* is introduced for making the sulphate of that metal.

*Caffea* (ground coffee) is added on account of its antidotal effect in opium poisoning.

*Canna* is an addition to the fecula.

*Ferri Sulphuretum* is introduced for making the sulphureted hydrogen, which is so frequently used as a test.

*Leptandra* is a remedy for trial. It is an emetico-cathartic, and eclectics contend that it is a substitute for calomel in its action on the lower and upper part of the canal.

*Matico* is also on trial as an aromatic tonic and stimulant. It possesses no astringent properties.

*Nectandra* has the properties of being antiperiodic and febrifuge. Its active principle is biberine, and is used in the form of a sulphate; it does not excite the circulation or the nervous system like quinine.

*Sodæ Sulphis* (sulphite of soda) is introduced as a convenient form of applying sulphurous acid in various cutaneous affections, where the pure acid is too irritating.

*Syrupus Fuscus* (molasses) is made officinal that it may be written for.

This completes all the new introductions into the primary list.

SECONDARY LIST.

*Achillea* (yarrow) is an antiperiodic and tonic. On trial.

*Angelica* is the root of the Angelica Archangelica, and not of the Angelica Atropurpurea.

*Berberis* (barberry), root of the berberis vulgaris, is a tonic and purgative.

*Brayera* (kousso) is the celebrated remedy for tape-worm. It is given in powder after a purgative, and while fasting.

*Delphinium* (larkspur), seed changed for the root.

*Enonymus* (wahoo), on trial as a diuretic and tonic. It destroys vermin in the hair effectually.

*Gelseminium* is an indigenous plant, and is said to be an energetic nervous tonic without astringent, nauseating, or purgative properties. There is claimed for it also the property of reducing the force and frequency of the pulse.

*Gossypii radix* (cotton root) is intended as a substitute for ergot.

*Hydrastis* possesses energetic tonic properties.

*Rottelera* (kameela) is a cathartic vermifuge, and may be used in lieu of kousso or pumpkin seed in tape-worm.

*Scutellaria* (scull cap) is an antispasmodic, and is considered as a specific for chorea. It is used in infusion.

ACETA.—*Acetum Lobeliæ* is a preparation which is the most convenient for preserving the active principles of the plant. The same may be said of *Acetum Sanguinariæ.*

*Acetum Opii* is changed in formula but not in strength.

ACIDA.—*Acidum Hydriodicum dilutum* is a valuable preparation of iodine when the stomach is irritable. *Acidum Muriaticum dilutum* is introduced for foot-baths, and should always be used fresh. *Acidium Phosphoricum dilutum* is a valuable nervous tonic. *Acidum Sulphurosum* (sulphurous acid diluted with water) is used as an application for the destruction of parasitic growths. *Acidum Valerianicum* is for making the valerianates.

Æ HERA.—*Æther Fortior* is the pure ether, and *Chloroformum purificatum* the pure chloroform, both of which are used for anæsthetic purposes.—*Am. Med. Times.*

